# Functional Outcomes at 6 and 12 Months Post-Injury in a Trauma Centre Population with Moderate-to-Severe Traumatic Injuries

**DOI:** 10.3390/jcm12165300

**Published:** 2023-08-15

**Authors:** Håkon Øgreid Moksnes, Christoph Schäfer, Mari Storli Rasmussen, Helene Lundgaard Soberg, Olav Røise, Audny Anke, Cecilie Røe, Pål Aksel Næss, Christine Gaarder, Eirik Helseth, Hilde Margrete Dahl, Morten Hestnes, Cathrine Brunborg, Nada Andelic, Torgeir Hellstrøm

**Affiliations:** 1 Department of Physical Medicine and Rehabilitation, Oslo University Hospital, P.O. Box 4956 Nydalen, N-0424 Oslo, Norway; christoph.schafer@unn.no (C.S.); masras@ous-hf.no (M.S.R.); uxheob@ous-hf.no (H.L.S.); ceroee@ous-hf.no (C.R.); nadand@ous-hf.no (N.A.); uxhetz@ous-hf.no (T.H.); 2 Institute of Health and Society, Research Centre for Habilitation and Rehabilitation Models & Services (CHARM), Faculty of Medicine, University of Oslo, P.O. Box 1072 Blindern, N-0316 Oslo, Norway; audny.anke@uit.no; 3 Department of Clinical Medicine, Faculty of Health Sciences, UiT the Arctic University of Norway, P.O. Box 6050 Langnes, N-9037 Tromsø, Norway; 4 Department of Rehabilitation, University Hospital of North Norway, P.O. Box 100, N-9038 Tromsø, Norway; 5 Faculty of Health Sciences, Oslo Metropolitan University, P.O. Box 4, St. Olavs Plass, N-0130 Oslo, Norway; 6 Norwegian Trauma Registry, Division of Orthopaedic Surgery, Oslo University Hospital, P.O. Box 4956 Nydalen, N-0424 Oslo, Norway; olav.roise@medisin.uio.no; 7 Institute of Clinical Medicine, Faculty of Medicine, University of Oslo, P.O. Box 1072 Blindern, N-0316 Oslo, Norway; paanae@ous-hf.no (P.A.N.); tingaa@ous-hf.no (C.G.); ehelseth@ous-hf.no (E.H.); uxhiah@ous-hf.no (H.M.D.); 8 Department of Traumatology, Oslo University Hospital, P.O. Box 4956 Nydalen, N-0424 Oslo, Norway; 9 Department of Neurosurgery, Oslo University Hospital, P.O. Box 4956 Nydalen, N-0424 Oslo, Norway; 10 Department of Child Neurology, Oslo University Hospital, P.O. Box 4956 Nydalen, N-0424 Oslo, Norway; 11 Division of Emergencies and Critical Care, Department of Research and Development, Oslo University Hospital, P.O. Box 4956 Nydalen, N-0424 Oslo, Norway; uxmohe@ous-hf.no; 12 Oslo University Hospital Trauma Registry, Oslo University Hospital, P.O. Box 4956 Nydalen, N-0424 Oslo, Norway; 13 Oslo Centre for Biostatistics and Epidemiology, Research Support Services, Oslo University Hospital, P.O. Box 4956 Nydalen, N-0424 Oslo, Norway; uxbruc@ous-hf.no

**Keywords:** traumatic injury, multiple trauma, rehabilitation, rehabilitation needs, functional outcome, prediction, GOSE

## Abstract

This study aims to evaluate the global functional outcomes after moderate-to-severe traumatic injury at 6 and 12 months and to examine the sociodemographic and injury-related factors that predict these outcomes. A prospective cohort study was conducted in which trauma patients of all ages with a New Injury Severity Score > 9 who were discharged alive from two regional trauma centres in Norway over a one-year period (2020) were included. The Glasgow Outcome Scale Extended (GOSE) score was used to analyse the functional outcomes. Regression analyses were performed to investigate the predictors of the GOSE score. Follow-up assessments were obtained from approximately 85% of the 601 included patients at both time points. The mean (SD) GOSE score was 6.1 (1.6) at 6 months and 6.4 (1.6) at 12 months, which corresponds to an upper-moderate disability. One-half of the patients had a persistent disability at 12 months post-injury. The statistically significant predictors of a low GOSE score at both time points were more pre-injury comorbidity, a higher number of injuries, and higher estimated rehabilitation needs, whereas a thorax injury with an Abbreviated Injury Scale ≥ 3 predicted higher GOSE scores. A high Glasgow Coma Scale score at admission predicted a higher GOSE score at 6 months. This study strengthens the evidence base for the functional outcomes and predictors in this population.

## 1. Introduction

Although traumatic injuries’ rate has declined [1], they continue to burden patients of all ages worldwide [2]. Traumatic injuries affect physical, behavioural, and psychosocial function; work ability; and health-related quality of life [3,4,5]. Despite an observed rise in functioning at the group level with time after injury [6], several studies demonstrated reduced function months to years after moderate and severe traumatic injury [3,7,8] across all age groups [9,10,11].

Evidence suggests that the coordination of an appropriate rehabilitation pathway can improve functional outcomes after trauma [12], especially after traumatic brain injury [13,14]. Unfortunately, knowledge about the effect of rehabilitation after other types of traumatic injuries is limited. Observational studies provided low-quality evidence in support of multidisciplinary intervention for individuals with multiple traumas [15]. In The Netherlands, integrated coordination between trauma care and rehabilitation and the rapid transfer of patients with multiple severe orthopaedic traumas to specialised rehabilitation resulted in a faster improvement in functional status compared with conventional trauma rehabilitation services [6]. To guide rehabilitation planning and provide prognostic information to patients and families, it is necessary to establish functional outcomes after injury and identify predictors of these outcomes. Previous studies provided important information about and described the functional outcomes for various trauma populations. Several studies report that poorer outcomes are associated with older age [3,5,16,17], more pre-injury comorbidity [3,5,16,18], and female sex [3,5,11,18,19]. In a study on patients hospitalised after injury, Polinder et al. demonstrated that older age, sex (female), more comorbidity, and the type of injury (spinal cord injury (SCI), hip fracture, or lower extremity injury) predicted a poor functional outcome at 9 and 24 months post-injury [3]. Gabbe et al. found that patients with an SCI or multiple traumas involving a head injury had decreased chances of good functional outcomes 24 months post-injury [5], whereas a higher Glasgow Coma Scale (GCS) predicted a better outcome in the population that sustained major trauma [20,21]. Few studies examined the global functional outcomes of the overall population that sustained moderate-to-severe injuries and the predictors of these outcomes. Furthermore, there is a lack of knowledge on how geographical factors influence functional outcomes in the trauma population [22]. Conducting population-based research may enhance the comprehension and prediction of outcomes following traumatic injuries, leading to a more coordinated rehabilitation process.

This study aimed to evaluate global functional outcomes at 6 and 12 months post-injury in a trauma centre population with moderate-to-severe traumatic injuries. Additional aims were to examine sociodemographic factors, including centrality of living, as well as other baseline factors that may predict these outcomes. 

## 2. Materials and Methods

### 2.1. Setting and Participants

This multicentre, prospective cohort study included patients of all ages with moderate-to-severe traumatic injuries admitted to the regional trauma centres of southeastern and northern Norway at Oslo University Hospital (OUH) and University Hospital of North Norway (UNN), respectively. OUH serves approximately 3 million people [23], and UNN serves approximately 482,000 people. This multicentre study was designed to encompass urban and rural areas across Norway, providing a more comprehensive representation of the trauma population and treatment chain. The healthcare system in Norway is publicly funded and provides universal access to hospital- and community-based care. The inclusion period was from January 2020 to December 2020 (OUH) and from February 2020 to January 2021 (UNN). The study protocol was published in 2021 [24]. Patient follow-ups were performed at 6 and 12 months post-injury.

### 2.2. Inclusion and Exclusion Criteria

The inclusion criterion was moderate-to-severe traumatic injury, defined as a New Injury Severity Score (NISS) > 9 according to the 2008 update of the 2005 Abbreviated Injury Scale (AIS) [25]. Patients of all ages were included if they also were admitted to the trauma centre within 72 h after the injury and had a hospital stay ≥2 days. A NISS score > 9 was chosen as the National Institute for Health and Care Excellence recommends that patients with an Injury Severity Score (ISS) > 9 in a trauma centre should be assessed for rehabilitation needs and receive a rehabilitation prescription if deemed necessary [26]. Exclusion criteria were non-Norwegian residents, death before discharge, and patients with insufficient knowledge of the Norwegian or English language.

### 2.3. Procedures

The procedure for inclusion was described in a previously published paper detailing the epidemiological characteristics of the included patients [27]. Patients were followed-up via structured interviews performed 6 and 12 months post-injury. 

### 2.4. Variables, Definitions, and Data Collection

#### 2.4.1. Primary Outcome Measure

The primary outcome was Glasgow Outcome Scale Extended (GOSE) rating at 6 and 12 months post-injury. The GOSE is a global measure of function that classifies the patient’s level of function on a scale: dead (GOSE = 1), vegetative state (GOSE = 2), dependent—need frequent help (lower severe disability; GOSE = 3), dependent—need some help (upper severe disability; GOSE = 4), unable to participate in one or more life roles (lower moderate disability; GOSE = 5), limited in one or more life roles (upper moderate disability; GOSE = 6), returned to normal life with some symptoms (lower good recovery; GOSE = 7), and fully returned to normal life (upper good recovery; GOSE = 8) [28,29]. The GOSE addresses the domains most likely to change in patients presenting with major trauma, such as self-care, mobility in the community, return to work, relationships, social activities, and leisure activities. The GOSE is a functional outcome measure with good responsiveness and a low ceiling effect when employed in major trauma populations with and without significant head injuries [20,30]. The original method of using the GOSE requires rating only the areas where a change has occurred; however, in the current study, we rated function without adjusting for pre-injury levels, as these were unknown. GOSE scores were obtained through structured clinical interviews at 6 and 12 months post-injury. For children, the harmonised GOSE was used [31], as the GOSE is the most common trauma-specific adult tool used in children [32]. In this study, we used the GOSE as a continuous variable, as the scores were normally distributed.

Patients who died during the follow-up period were identified through the medical journal system and assigned GOSE = 1.

#### 2.4.2. Predictors

Information available at baseline (sociodemographic, injury, and treatment characteristics) was used as a base for identifying possible predictors of functional outcomes at both 6 and 12 months, as this is relevant in a planning situation. 

##### Patient Characteristics

Sociodemographic factors, such as sex, age, and centrality of living area, were obtained from medical records and supplemented by the patient/caregiver. Age was treated as a continuous variable. For centrality, the Norwegian Centrality Index (NCI) was applied to the patients’ municipality of residency. The index classifies Norwegian municipalities into six classes (1 = most central, and 6 = least central) defined by the time of travel to workplaces and other official services [33]. For this study, the NCI was dichotomised into ‘central’ (NCI 1–2) and ‘less central’ (NCI 3–6). Pre-injury comorbidity, as classified by the American Society of Anesthesiologists (ASA)’s Physical Status Classification System [34], was also recorded and dichotomised (‘healthy’ = ASA 1, and ‘comorbidity’ = ASA 2–6).

##### Injury and Treatment Characteristics

Data on mechanism of injury, influence of substance use at the time of injury, work-related injury, and GCS at the time of admission to a local hospital or trauma centre were collected from medical records. GCS was recorded for all patients, including those without head injuries, and was treated as a continuous variable. The influence of substance use was determined based on laboratory tests (alcohol) or clinical suspicion by healthcare providers or patient reports. If no information was available, a ‘no/unknown value’ was assigned. Any type of non-surgical (e.g., respirator, physiotherapy, and psychiatric assessment) or surgical (e.g., invasive intracranial pressure measurement, chest tube drainage, coiling of abdominal bleeding, and fracture surgery) procedures and any type of significant medical complication (e.g., hydrocephalus, infection, and embolism) during the hospital stay were dichotomised into ‘no’/’yes’. Length of hospital stay was defined as the number of days spent in the acute care departments of the trauma centres. Data on injured body region, injury severity (AIS, NISS, and ISS), and number of injuries were extracted from the trauma registries of the two hospitals. NISS, ISS, and number of injuries were treated as continuous variables. Head, thorax, abdomen, spine, or extremity injury with AIS ≥ 3 was dichotomised. We defined a NISS score of 10–15 as ‘moderate’ and 16–75 as ‘severe’ for the purpose of predictor analysis.

The need for community-based rehabilitation and healthcare service delivery for the period 0–6 months post-injury was estimated using the Needs and Provision Complexity Scale (NPCS) Clinician version, a 15-item measure with a total range of 0–50. The NPCS consists of two main domains: ‘Health and personal care needs’ (score range 0–25), with subscales ‘Health care’, ‘Personal care’, and ‘Rehabilitation’; ‘Social care and support needs’ (score range 0–25), which includes subscales ‘Social and family support’, and ‘Environment’ [35]. In the current project, specialists in rehabilitation medicine (N.A., C.S., and H.M.) estimated these needs at discharge from the trauma centres using medical records, clinical experience, and research evidence. The inter-rater reliability between these three rehabilitation specialists, calculated with intra-class correlations (ICC), was good for consistency (ICC 0.858) and absolute agreement (ICC 0.756). The calculation was based on a sample of 11 patients. NPCS Needs scores were treated as continuous variables.

### 2.5. Statistical Analysis

Descriptive statistics are presented as frequencies with percentages, mean with standard deviation (SD), or median with interquartile range (IQR). Dropout analyses according to differences in sex, age, and injury severity were performed using Student’s t-test and the chi-square test, as appropriate. Uni- and multivariable linear regression analyses were performed to investigate predictors of GOSE scores at 6 and 12 months post-injury. Possible predictors of outcomes were selected based on univariable analyses (with a cut-off criterion of *p* = 0.1), published studies, and clinical experience. Linear regression was chosen due to the distribution of the GOSE scores. Normality of residuals were examined by plotting residuals against fitted values and found to be satisfied. The following factors were included: sex; age at the time of injury; NCI; pre-injury ASA; the influence of substance use at the time of injury; NISS; number of injuries; head, thorax, abdominal, spine, or extremity injury with AIS ≥ 3; GCS at time of admission to the local hospital or trauma centre; sum score of NPCS Needs 0–6 months post-injury (estimated at baseline). We present the full multivariable model to demonstrate which factors most strongly predicted GOSE score at 6 and 12 months post-injury when considered together, without subsequent elimination of variables driven by our data. Only complete case analyses were performed for multiple regression analyses, as the overall proportion of missing values was very low (0.6–0.8%). Consequently, multiple imputations were not performed, as this would most likely not influence the statistical analyses. The data from the two centres were merged, and the analysis was performed for the whole cohort. The results are presented as regression coefficients (B) with 95% confidence intervals (CI) and explained variance (R^2^). The degree of multi-collinearity between the independent variables was examined using Spearman’s correlation coefficient ≥0.7 as a cut-off.

Statistical significance was set at 0.05, and the *p*-values were two-tailed. SPSS statistics version 28 (IBM Corp., Armonk, NY, USA) was used for the statistical analyses.

## 3. Results

### 3.1. Population and Injury Features

**Figure 1:**
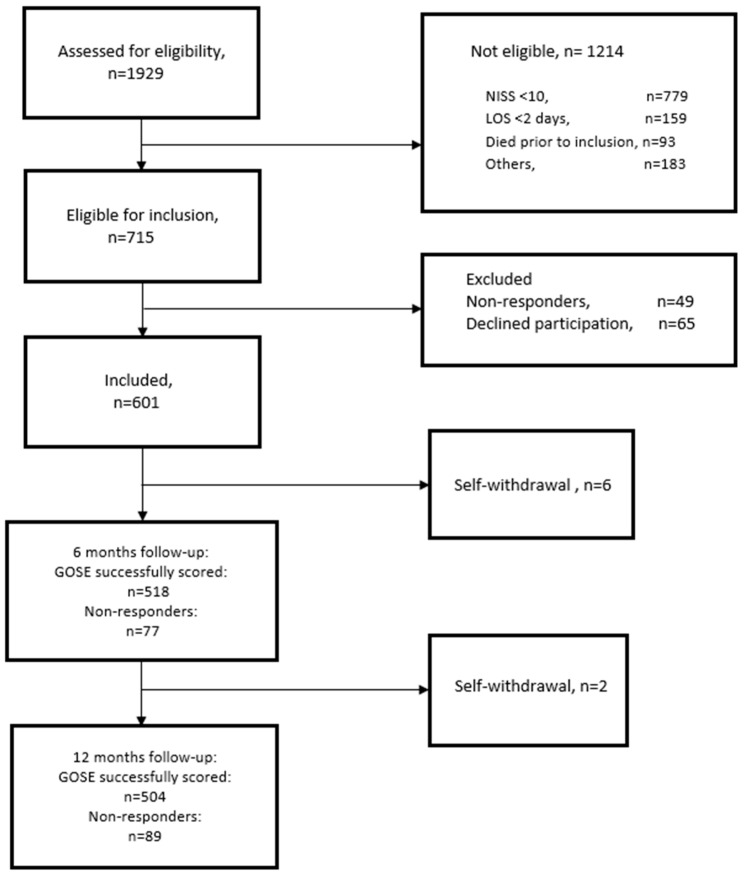
Flowchart. GOSE, Glasgow Outcome Scale Extended; LOS: length of hospital stay; NISS, New Injury Severity Score.

In total, 715 patients were eligible for inclusion in the study, and 601 (554 at OUS and 47 at UNN) were successfully contacted and consented to participate. Figure 1 presents the details of the recruitment process and study flow. The GOSE was successfully scored for 518 patients (86.2% of the total) at 6 months post-injury and 504 patients (83.9% of the total) at 12 months post-injury. 

Table 1 presents the sociodemographic and injury-related data collected at baseline. Most patients were male (75%), the mean (SD) age of the patients was 47 (21.2) years, and 63 patients were children/adolescents (age < 18 years at the time of injury). The median (IQR) NISS was 22 (16, 29), and the median ISS was 17 (10, 24), which corresponded to severe injury. Two separate dropout analyses were performed. (1) At inclusion: the analysis revealed that the mean age of the dropout group (n = 114) was somewhat higher (51 (SD: 23.1) years) and the proportion of males was lower (74%) than in the inclusion group; however, the differences were not significant. (2) At follow-up: the mean age (at the time of injury) for non-responders at 12 months follow-up (n = 97) was somewhat lower than for responders (n = 504) (43 (SD: 18.6) years vs. 48 (SD: 21.6) years, *p* = 0.05). The differences in the proportion of males (74.2% vs. 75.2%) and severe injury (76.3% vs. 76.0%) were not significant.

### 3.2. Outcomes

**Figure 2:**
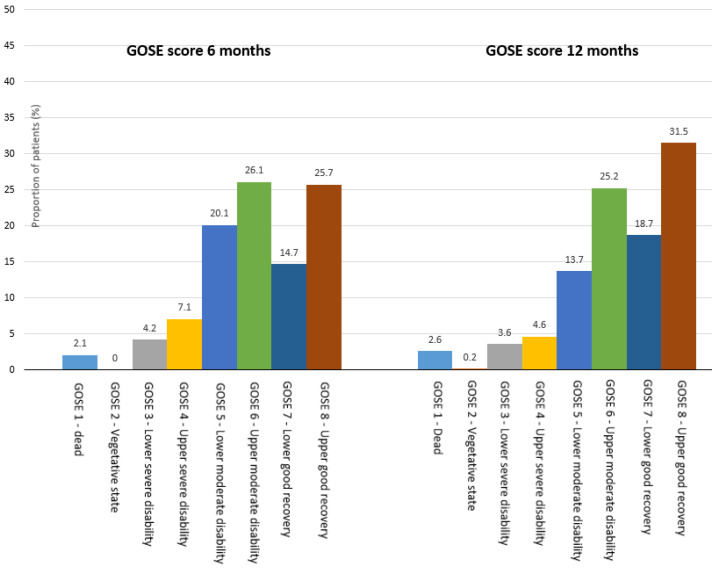
GOSE scores at 6 and 12 months post-injury, with proportion of patients in categories. GOSE, Glasgow Outcome Scale Extended.

The primary outcome was the GOSE score at 6 and 12 months post-injury (Figure 2). During the 12-month follow-up period, there were 14 (2.2% of the cohort) post-discharge deaths; most deaths (n = 11, 85%) occurred within 6 months post-discharge. Only one patient was reported to be in a vegetative state at the end of the follow-up period (12 months).

The mean (SD) GOSE scores were 6.1 (1.6) at 6 months and 6.4 (1.6) at 12 months, which corresponded to an upper-moderate disability.

### 3.3. Predictors of GOSE Score at 6 and 12 Months

Results from the 6-month prediction of the GOSE score (Table 2) demonstrated that an ASA score ≥ 2 predicted a lower GOSE score. Furthermore, an increase in the number of injuries or increased estimated NPCS Needs 0–6 months score were negative predictors of the GOSE score. A thorax injury with an AIS ≥ 3 or a high GCS at admission were significant positive predictors of the GOSE score. The GOSE scores at 12 months (Table 2) suggest that the significant predictors from the 6-month analysis were still present at 12 months, except for GCS at admission. As sensitivity analyses, the multivariable models were replicated, excluding those who died between baseline and the 6-month follow-up. The results did not significantly differ from the models including those who died (data not shown). 

The factors included in the model explained 36% of the variation in the GOSE score (R^2^ = 0.36) at 6 months and 32% of the variation (R^2^ = 0.32) at 12 months.

## 4. Discussion

At 12 months post-injury, one-half of the patients presented with a persistent disability. The most important predictors of global outcome were pre-injury comorbidity, the number of injuries, the presence of a severe thorax injury, and estimated rehabilitation needs, indicating that these factors should be considered when planning rehabilitation services for this population.

Comparing our findings with others is challenging due to inconsistent inclusion criteria, inconsistent follow-up durations, and variations in how the GOSE is operated. Furthermore, longitudinal studies comprising injured patients of all ages are scarce.

### 4.1. Functional Outcome at 6 and 12 Months

Our study found that only 2.2% of the cohort died during post-discharge follow-up, with just one patient being in a vegetative state after 12 months. In an Australian study, Mitra et al. examined patients of all ages with an ISS > 15 who required urgent surgery or intensive care unit admission and received a massive transfusion. They had comparable results, as 1.4% of the population died within the first 12 months after discharge, and 0.6% were in a vegetative state at follow-up [20]. In a study in Cameroon on patients discharged after hospital treatment for a traumatic injury, Ding et al. reported that 5.4% died within 6 months [36]. Their population was considerably younger than that of the present study. Even if we only consider the adults in our study, the proportion of patients who died in the first 12 months is low (2.4%). Caution should be taken when comparing the proportion of patients registered with a GOSE score of 1 (dead) between studies. This is because deceased individuals are typically identified through either a register or a medical record system that promptly and accurately records deaths, while information about surviving patients may be lost due to loss to follow-up. As a result, there may be an overestimation of the proportion of deceased individuals. This bias affects all the proportions of the GOSE scores, particularly in cases with high rates of lost-to-follow-up participants.

At 6 months, the proportion of individuals with a severe disability (GOSE = 3–4) in our study was lower (11%) than in Rainer et al.’s report (19%) [37]. However, Rainer et al.’s study in Hong Kong only included adults, and other differences in inclusion criteria and follow-up rates make comparison difficult. Ding et al. included patients of all ages and reported a higher proportion with a severe disability (22%) than in our study. In comparison, Gabbe et al.’s study (2012) from Australia focused on discharged adult major trauma patients at 12 months post-injury and found that the proportion of patients with a severe disability was higher (19%) than in our findings (8%). They reported that 74% were living independently (GOSE = 5–8) 12 months post-injury [21], compared with 91% in our study population. Furthermore, they adjusted the GOSE score for pre-injury function level, which might have led to higher scores compared with our method; therefore, the differences in the results are likely even greater.

Ding et al. reported good recovery (GOSE = 7–8) for 70% of patients at 6 months [36], a more favourable result than the 40% reported in our study. It is possible that factors other than the study design, such as sociodemographic differences, contributed to these variations in results. At 12 months, Gabbe et al. (2012) reported a lower percentage of good recovery (35%) in their cohort [21] compared to our finding of 51%. Variations in inclusion criteria and participants’ attrition rates as well as potential disparities in rehabilitation services may explain these observed differences.

### 4.2. Predictors of GOSE

#### 4.2.1. Sociodemographic Factors

Several studies demonstrated poorer functional outcomes with increasing age at the time of injury [16,17,20,36]. These findings were echoed in the multivariable analysis in our study, although the result was not significant. We did not find the influence of female sex as an independent predictor of lower functional outcome after trauma, in contrast to previous studies [19,36]. Mitra et al. also reported this lack of association [20]. However, Rainer et al. reported female sex as a predictor of lower functional outcome at 12 months but not at 6 months [38]. Pre-injury comorbidity, assessed by the ASA, negatively predicted functional outcomes at 6 and 12 months. This negative impact was demonstrated in previous studies [3,16,18,21,37], highlighting the importance of assessing baseline conditions when evaluating post-traumatic outcomes and rehabilitation. Comorbidity should also be included in the standardised set of the determinants of functional outcome after major trauma.

Few studies explored the impact of geography on functional outcomes after trauma. Such an impact could be relevant when planning rehabilitation services. However, in this study investigating the overall outcome after trauma, no evidence was found for geographic variation in functional outcomes.

#### 4.2.2. Injury-Related Factors

A higher number of injuries predicted lower functional outcomes at 6 and 12 months in our study. This aspect of traumatic injuries has not been extensively studied. Results vary in studies that have examined severe injury as a predictor of reduced functional outcome; Mitra et al. suggested worse [20], while Holtslag et al. found no association [16] between injury severity and functional outcomes. Having multiple injuries, including both head and orthopaedic injuries, is likely to have a greater impact on various functional aspects compared to isolated injuries. This makes it more challenging for patients to adapt to and compensate for any functional losses they may experience. Further, clinical observations indicate that orthopaedic injuries affecting the extremities and the spine significantly influence functional outcomes. Many of these injuries are classified as having an AIS < 3.

Considering severe injuries to multiple body regions, the only statistically significant predictor of function was found to be a thorax injury with an AIS ≥ 3, which was a predictor of a more positive outcome. In line with this, Gabbe et al. (2012) reported that chest or abdominal injuries in isolation were positive predictors of a higher GOSE score at 12 months when compared to an isolated head injury [21] and that patients with isolated chest and abdominal injuries had high levels of recovery early in the post-injury phase (2016) [5]. In a narrative literature review by Baker et al. [39], the authors highlighted the paucity of data related to long-term outcomes after thoracic injuries. They reported that symptoms related to thoracic injuries improve over time, although the sequelae impact all aspects of daily functioning. However, based on clinical experience, it is observed that thorax injuries with an AIS < 3 may have a greater influence on outcomes for patients who are discharged alive, compared to thorax injuries with an AIS ≥ 3.

Another positive predictor of outcomes at 6 months was a high GCS score at the time of admission to a local hospital or trauma centre, which is consistent with the findings of Mitra et al. and Rainer et. al. These studies demonstrated that high GCS scores predicted better functional outcomes at 6 and 12 months following major trauma [20,38]. Additionally, Gabbe et al. (2012) also identified a higher GCS score as a positive predictor of functional outcome 12 months post-injury [21]. Although the association was not statistically significant at 12 months in our study, the consistent findings across multiple studies suggest that a higher GCS score at admission is generally associated with better functional outcomes after major trauma.

To our knowledge, no other studies included rehabilitation needs as an independent variable when analysing functional outcomes after trauma. We found that higher estimated needs for community-based rehabilitation in the first 6 months post-injury predicted a lower GOSE score at both time points. The estimated NPCS Needs value incorporates injury severity and complexity, as assessed by a rehabilitation specialist, and may reflect aspects beyond the AIS/NISS/ISS scores, which are mainly used to reflect the impact of injuries on the risk of death in the acute phase [25].

Even though this study identifies the predictors of worse outcomes as assessed by global functioning, it does not answer how to best intervene to maximize functional outcomes. Our findings, however, support the need to establish a strategy to specifically target patients with a higher level of pre-injury comorbidity, a higher number of injuries, and higher estimated rehabilitation needs. It is important to note that there is, currently, limited knowledge regarding rehabilitation needs following trauma [40,41]. A recent systematic review on service provision revealed variations in the rehabilitation and complexities experienced by patients after trauma events [40].

In a recent study on adherence to the guidelines in the Norwegian trauma plan [42,43], we observed that the initiation of early rehabilitation treatment for patients in Norwegian trauma centres occurred later, compared to other studies. Moreover, there was limited assessment by a rehabilitation physician within three days after admission, and only a small proportion of participants were directly transferred from the trauma centre to specialised rehabilitation. Although direct transfer was common for patients with a spinal cord injury, it was less frequent for those with a severe head injury, and priority was rarely given to patients with severe extremity injuries. These findings highlight the need for a more comprehensive integration of rehabilitation into the acute treatment phase after trauma.

The assessment of community-based rehabilitation needs (NPCS) in our study was previously used to assess unmet community-based healthcare and rehabilitation needs in long-term neurological conditions [44]. That study revealed significant unmet needs in the subdomains of rehabilitation, social support, and equipment, which aligns with our observations in the trauma follow-up clinical practice. Thus, there is a need for in-depth analyses of the unmet rehabilitation needs for community-based rehabilitation following trauma, which will be accomplished in our upcoming paper.

### 4.3. Strengths and Limitations

The strengths of this study are its multicentre, prospective design and its coverage of a substantial proportion (>60%) of the Norwegian trauma centre population. The sample size included patients from all age groups, and patients were not excluded based on comorbidity. There was a low level of dropouts and missing data, which ensured a robust analysis.

The relatively short 12-month follow-up period is a limitation of this study. Selection bias may have been introduced at inclusion and during follow-up in this observational study. However, the number of non-responders or those lost to follow-up was low.

This study was performed during the COVID-19 pandemic, which could have influenced factors such as inclusion in the study, services received, and physical and mental health, potentially impacting the functional outcomes. In addition, the main instrument (GOSE) has several limitations, as some aspects of the scale are only relevant if the patient has returned to the community [45]. Other instruments, such as the GOSE Pediatric and King’s Outcome Scale for Childhood Head Injury, were recommended for children [46].

We did not control for pre-injury functioning or health incidents unrelated to the injury in the follow-up period. This may have reduced the comparability of our study with other studies and may have limited our understanding of the significance of the impact of the injury on post-injury function level. Despite this limitation, unadjusted outcomes still provide valuable information when planning services. The use of the GOSE as the only major outcome measure may have been a limitation. However, this paper is part of a larger trauma project that encompasses rehabilitation needs. In planned future papers, we will evaluate and discuss symptom burden, psychosocial function, and health-related quality of life.

Differences in healthcare delivery and funding for trauma patients between countries should be considered when interpreting the findings, and such differences can reduce the generalisability of the results. Future studies should include a broader trauma population, such as those who have been treated without referral to the trauma centre, and it is also important to examine the population with minor injuries. The cohort of this study represents the whole population, with an age range of 0–92 years; a future publication will further describe the functional outcomes for the paediatric population.

## 5. Conclusions

In this study, one-half of the patients experienced a persistent disability in the first 12 months after moderate-to-severe traumatic injury. This study strengthens the evidence for pre-injury comorbidity, various aspects of injury severity, and estimated rehabilitation needs as important predictors of global functional outcomes. Patient populations with these characteristics should be targeted for more extensive follow-up and rehabilitation services to help improve outcomes following injury.

## Figures and Tables

**Table 1:** Sociodemographic and injury-related factors (baseline).

Characteristics	Baseline
Total n (%)	601 (100)
Sex n (%) Male Female	451 (75.0) 150 (25.0)
Age (years), mean (SD)	46.88 (21.2)
Centrality Index (NCI) n (%) Category 1–2 (central) Category 3–6 (less central)	337 (56.1) 264 (43.9)
Pre-injury comorbidity n (%) Healthy = ASA 1 Comorbidity = ASA 2–4	327 (54.4) 274 (45.6)
Injury mechanism n (%) Falls Transport-related Violence Others	243 (40.4) 227 (37.8) 18 (3.0) 113 (18.8)
Influence of substance at the time of injury n (%) No/unknown Yes	468 (77.7) 133 (22.3)
Work-related injury ^1^ n (%) No Yes	557 (93.0) 42 (7.0)
Injury severity New Injury Severity Score (NISS) Median (IQR) Injury Severity Score (ISS) Median (IQR)	22 (16, 29) 17 (10, 24)
Number of injuries Median (IQR)	5 (3, 8)
Body region with AIS ≥ 3 n (%) Head Thorax Abdomen Spine Extremities, upper and lower External and other	250 (41.6) 197 (32.8) 73 (12.1) 80 (13.3) 98 (16.3) 2 (0.3)
GCS at time of admission to local hospital or trauma centre ^2^ Median (IQR)	15 (13, 15)
Surgical procedures during hospital stay n (%) No Yes	214 (35.6) 387 (64.4)
Non-surgical procedures during hospital stay n (%) No Yes	94 (15.6) 507 (84.4)
Treatment complications during hospital stay n (%)	188 (31.3)
Length of hospital stay n (%) (acute care unit at the trauma centre), days Median (IQR)	6 (3, 10)
Sum score NPCS Needs Mean (SD)	10.3 (6.0)

SD, standard deviation; NCI, Norwegian Centrality Index; ASA, American Society of Anesthesiologists classification; NISS, New Injury Severity Score; IQR, interquartile range; ISS, Injury Severity Score; AIS, Abbreviated Injury Scale; GCS, Glasgow Coma Scale; NPCS, Needs and Provision Complexity Scale 0–6 months post-injury, estimated at baseline. ^1^: Missing = 2. ^2^: Missing = 3.

**Table 2:** Sociodemographic, injury-, and treatment-related predictors of GOSE score at 6 and 12 months post-injury.

		Outcome Variable: GOSE Score at 6 Months n = 518 ^1^						Outcome Variable: GOSE Score at 12 Months n = 504 ^2^					
		Univariable Regression			Multivariable Regression			Univariable Regression			Multivariable Regression		
		B	95% CI	*p*-Value	B	95% CI	*p*-Value	B	95% CI	*p*-Value	B	95% CI	*p*-Value
Sociodemographic characteristics	Sex												
	Male	(ref.)			(ref.)			(ref.)			(ref.)		
	Female	−0.247	(−0.57, 0.08)	0.14	0.017	(−0.25, 0.29)	0.86	−0.31	(−0.64, 0.02)	0.07	−0.064	(−0.35, 0.22)	0.65
	Age at time of injury (years)	**−0.013**	**(−0.02, −0.01)**	**<0.001**	−0.006	(−0.01, 0.00)	0.07	**−0.015**	**(−0.02, −0.01)**	**<0.001**	−0.006	(−0.01, 0.00)	0.11
	Centrality index (NCI)												
	1–2 (central)	(ref.)			(ref.)			(ref.)			(ref.)		
	3–6 (less central)	−0.041	(−0.32, 0.24)	0.77	0.150	(−0.09, 0.38)	0.21	−0.150	(−0.44, 0.14)	0.31	0.069	(−0.18, 0.32)	0.59
	Pre-injury comorbidity												
	ASA 1 (healthy)	(ref.)			(ref.)			(ref.)			(ref.)		
	ASA ≥ 2 (comorbidity)	**−0.924**	**(−1.19, −0.66)**	**<0.001**	**−0.538**	**(−0.83, −0.25)**	**<0.001**	**−1.002**	**(−1.28, −0.73)**	**<0.001**	**−0.673**	**(−0.98, −0.37)**	**<0.001**
Injury- and treatment-related characteristics	Influence of substance at the time of injury	−0.222	(−0.56, 0.12)	0.20	−0.106	(−0.40, 0.19)	0.48	−0.113	(−0.47, 0.24)	0.53	0.068	(−0.25, 0.38)	0.67
	Injury severity (NISS)												
	Moderate injury (NISS 10–15)	(ref.)			(ref.)			(ref.)			(ref.)		
	Severe injury (NISS > 15)	**−0.521**	**(−0.85, −0.20)**	**0.002**	−0.056	(−0.37, 0.26)	0.73	**−0.459**	**(−0.79, −0.13)**	**0.01**	−0.047	(−0.38, 0.29)	0.78
	Number of injuries	**−0.125**	**(−0.16, −0.09)**	**0.001**	**−0.074**	**(−0.11, −0.04)**	**<0.001**	**−0.107**	**(−0.15, −0.07)**	**<0.001**	**−0.061**	**(−0.10, −0.02)**	**0.002**
	Body regions with AIS ≥ 3												
	Head	**−0.555**	**(−0.83, −0.28)**	**<0.001**	0.100	(−0.21, 0.42)	0.53	**−0.503**	**(−0.79, −0.22)**	**<0.001**	0.128	(−0.21, 0.46)	0.45
	Thorax	0.106	(−0.19, 0.40)	0.48	**0.427**	**(0.13, 0.72)**	**0.01**	0.117	(−0.19, 0.42)	0.45	**0.372**	**(0.06, 0.69)**	**0.02**
	Abdomen	**0.547**	**(0.13, 0.97)**	**0.01**	−0.034	(−0.41, 0.34)	0.86	**0.609**	**(0.15, 1.07)**	**0.01**	0.107	(−0.32, 0.53)	0.62
	Spine	**−0.424**	**(−0.82, −0.03)**	**0.03**	−0.117	(−0.48, 0.25)	0.53	**−0.628**	**(−1.03, −0.23)**	**0.002**	−0.223	(−0.61, 0.16)	0.26
	Extremity	**−0.675**	**(−1.05, −0.30)**	**<0.001**	−0.245	(−0.60, 0.11)	0.17	−0.208	(−0.62, 0.20)	0.32	0.112	(−0.27, 0.50)	0.57
	GCS at time of admission to local hospital or trauma centre	**0.129**	**(0.09, 0.17)**	**<0.001**	**0.045**	**(0.01, 0.09)**	**0.03**	**0.114**	**(0.07, 0.15)**	**<0.001**	0.037	(−0.01, 0.08)	0.09
	Estimated rehabilitation needs Sum score NPCS Needs (0–6 months)	**−0.145**	**(−0.17, −0.12)**	**<0.001**	**−0.108**	**(−0.13, −0.08)**	**<0.001**	**−0.134**	**(−0.16, −0.11)**	**<0.001**	**−0.101**	**(−0.13, −0.08)**	**<0.001**

GOSE, Glasgow Outcome Scale Extended; B, regression coefficient; CI, confidence interval; NCI, Norwegian Centrality Index; ASA, American Society of Anesthesiologists classification; NISS, New Injury Severity Score; AIS, Abbreviated Injury Scale; GCS, Glasgow Coma Scale; NPCS, Needs and Provision Complexity Scale. Significant results are presented in bold. Six months: R^2^ = 0.36. Twelve months: R^2^ = 0.32. ^1^. Responders: n = 518. In the multivariable regression analysis, two patients were removed due to missing data (n = 516). ^2^. Responders: n = 504. In the multivariable regression analysis, two patients were removed due to missing data (n = 502).

## Data Availability

The datasets generated and/or analysed in the current study are not publicly available due to the sensitivity of the material.
